# Editorial: Emergency in psychiatry—The various facets of behavioral emergencies, crises and suicidality, volume II

**DOI:** 10.3389/fpsyt.2022.1121865

**Published:** 2022-12-23

**Authors:** Johannes M. Hennings, Ksenija Slankamenac

**Affiliations:** ^1^Department of Dialectical Behavioral Therapy, kbo Clinic Region Munich, Munich, Germany; ^2^Institute of Emergency Medicine, University Hospital Zürich, Zurich, Switzerland

**Keywords:** emergency psychiatry, suicidality, coercive, forced medication, emergency medical service (EMS), suicide prevention, HPA axis, suicide attempt

This second volume of our Research Topic ties in with the previous one spotlighting most important issues in emergency psychiatry: suicidality, coercive measures and coping of psychological challenges for health care providers.

Suicidality is without doubt one of the most alerting and urgent symptomatology in mental health. More than 700,000 people worldwide die by suicide every year, and the number of suicide attempts (SA) is estimated as 20-times higher (1). While risk factors for suicidal behavior such as childhood maltreatment, non-suicidal self-injury, and previous SA have been identified across psychiatric disorders (2, 3), up to 70% of suicides have been linked to affective disorders (4). Since the knowledge of suicide predictors has shown limited effect reducing suicide risk in clinical practice over the years according to a recent meta-analysis (5), we still need to improve our understanding of the neurobiology, contributing psychosocial factors and efficient clinical approaches of suicidality. With respect to these needs, dysregulation of the stress hormone system, namely the hypothalamic-pituitary-adrenocortical (HPA) axis, is one of the most consistent neurobiological findings in both, major depression and suicidality. Interestingly, Hennings et al. show that previous SA—which is also an important clinical suicide risk factor—not only have an attenuating effect on the HPA axis activity during a depressive episode but also impact on the recovery of altered HPA axis reactivity due to depression indicating both, acute effects on HPA axis as well as persistent alteration on stress hormone regulation related to a history of suicide attempts.

Chronic pain as well as serious physical conditions associated with somatic disorders like cancer are well known contributors to suicidality (6, 7). But also, psychosocial stress of people living with HIV can induce suicide ideations as shown in the study of Wang et al. Further stressing the need of effective suicide prevention and in line with a world-wide phenomenon, Li et al. describe in a large dataset of North China that most suicide attempters admitted to hospital were relatively young (15–34 years) in both, urban and rural areas. While pesticide self-poisoning accounts for about one-third of the world's suicides (8), it is further documented in this study that in rural areas ingestion of lethal pesticides is still the leading cause (52.1%) suggesting that regulatory restrictions on the accessibility of these poisons would be an promising and easy mean to prevent a significant number of SA and suicides. Given the fact that suicide rates after discharge from psychiatric facilities is about 100 times the global suicide rate during the first 3 months after discharge, and that patients admitted with suicidal thoughts or behaviors had rates near 200 times the global rate in this period of time (9), knowledge on the necessity of immediate follow-up appointments after discharge of patients hospitalized due to suicidality (according to national guideline recommendation for the treatment of suicidal depressed patients) was alarmingly low in a study of German psychotherapists (Teismann et al.).

Despite significant negative impact on mental health including the development of symptoms of PTSD (10, 11) but poor scientific evidence for its general practical and clinical effectiveness (12–14), coercive means are still widely used in psychiatry and even mental health care providers are convinced that their application is inevitable in many cases (15). The Recovery-orientated psychiatric care concept “Weddinger Modell” was initiated to reduce coercive measures in acute psychiatric settings (16). Interestingly, while it has been shown to reduce mechanical coercive measures (17) and post-coercion PTSD symptoms (18), Czernin et al. now demonstrate that in the same time incidence of forced medication did not increase but that the maximum doses of psychiatric drugs needed for acute symptom control can even be reduced including maximum forced haloperidol dose. These findings support the development of a more participating and recovery-orientated psychiatric care also in acute settings and may improve the public recognition of mental health care provided by psychiatric hospitals and their acceptance of the patients.

Although the emotional distress occurring in health professionals while being confronted to coercive means (19), as suicide first responders (20) or in medical emergencies in general intuitively has a psychological impact, mental health in health professionals is a dramatically under-investigated field. As shown in the review of Abdulaziz Alghamdi for disaster emergency medicine services including those occupied in terror attacks, earthquake and COVID-19 pandemics, health care providers can significantly suffer from anxiety and affective disorders as well as PTSD symptoms. Indeed, they themselves need professional support in many cases. Modern technologies like app-based programs (21, 22) my provide a low-threshold opportunity to address affected individuals or individuals at risk to develop serious mental health issues.

## Author contributions

JH wrote the first draft of the manuscript. Both authors contributed to manuscript revision, read, and approved the submitted version.

## References

[1] World Health Organization. Suicide Prevention. WHO (2020).

[2] Klonsky ED May AM Glenn CR. The relationship between nonsuicidal self-injury and attempted suicide: converging evidence from four samples. J Abnorm Psychol. (2013) 122:231–7. 10.1037/a0030278 23067259

[3] Franklin JC Ribeiro JD Fox KR Bentley KH Kleiman EM Huang X. Risk factors for suicidal thoughts and behaviors: a meta-analysis of 50 years of research. Psychol Bull. (2017) 143:187–232. 10.1037/bul0000084 27841450

[4] Rihmer Z Kiss K. Bipolar disorders and suicidal behaviour. Bipolar Disord. (2002) 4 Suppl 1:21–5. 10.1034/j.1399-5618.4.s1.3.x 12479671

[5] Carroll R Metcalfe C Gunnell D. Hospital presenting self-harm and risk of fatal and non-fatal repetition: systematic review and meta-analysis. PLoS ONE. (2014) 9:e89944. 10.1371/journal.pone.0089944 24587141 PMC3938547

[6] McFarland DC Walsh L Napolitano S Morita J Jaiswal R. Suicide in patients with cancer: identifying the risk factors. Oncology. (2019) 33:221–6. 31219606

[7] Racine M. Chronic pain and suicide risk: a comprehensive review. Prog Neuropsychopharmacol Biol Psychiatry. (2018) 87(Pt B):269–80. 10.1016/j.pnpbp.2017.08.020 28847525

[8] Gunnell D Eddleston M Phillips MR Konradsen F. The global distribution of fatal pesticide self-poisoning: systematic review. BMC Public Health. (2007) 7:357. 10.1186/1471-2458-7-357 18154668 PMC2262093

[9] Chung DT Ryan CJ Hadzi-Pavlovic D Singh SP Stanton C Large MM. Suicide rates after discharge from psychiatric facilities. JAMA Psychiatry. (2017) 74:694–702. 10.1001/jamapsychiatry.2017.1044 28564699 PMC5710249

[10] Frueh BC Knapp RG Cusack KJ Grubaugh AL Sauvageot JA Cousins VC. Patients' reports of traumatic or harmful experiences within the psychiatric setting. Psychiatr Serv. (2005) 56:1123–33. 10.1176/appi.ps.56.9.1123 16148328

[11] Fugger G Gleiss A Baldinger P Strnad A Kasper S Frey R. Psychiatric patients' perception of physical restraint. Acta Psychiatr Scand. (2016) 133:221–31. 10.1111/acps.12501 26472265

[12] Huber CG Schneeberger AR Kowalinski E Fröhlich D von Felten S Walter M. Suicide risk and absconding in psychiatric hospitals with and without open door policies: a 15 year, observational study. Lancet Psychiatry. (2016) 3:842–9. 10.1016/S2215-0366(16)30168-7 27477886

[13] Kisely SR Campbell LA O'Reilly R. Compulsory community and involuntary outpatient treatment for people with severe mental disorders. Cochrane Database Syst Rev. (2017). Available online at: https://www.cochranelibrary.com/cdsr/doi/10.1002/14651858.CD004408.pub5/full (accessed December 7, 2022). 10.1002/14651858.CD004408.pub5 PMC6464695 28303578

[14] Muralidharan S Fenton M. Containment strategies for people with serious mental illness. Cochrane Database Syst Rev. (2006) 1–15. 10.1002/14651858.CD002084.pub2 16855984 PMC11384505

[15] Morandi S Silva B Mendez Rubio M Bonsack C Golay P. Mental health professionals' feelings and attitudes towards coercion. Int J Law Psychiatry. (2021) 74:101665. 10.1016/j.ijlp.2020.101665 33401095

[16] Wullschleger A Vandamme A Ried J Pluta M Montag C Mahler L. Standardized debriefing of coercive measures on psychiatric acute wards: a pilot study. Psychiatr Prax. (2019) 46:128–34. 10.1055/a-0651-6812 30253421

[17] Czernin K Bermpohl F Heinz A Wullschleger A Mahler L. Effects of the psychiatric care concept “Weddinger Modell” on mechanical coercive measures. Psychiatr Prax. (2020) 47:242–8. 10.1055/a-1116-0720 32198733

[18] Wullschleger A Vandamme A Mielau J Renner L Bermpohl F Heinz A. Effect of standardized post-coercion review session on symptoms of PTSD: results from a randomized controlled trial. Eur Arch Psychiatry Clin Neurosci. (2021) 271:1077–87. 10.1007/s00406-020-01215-x 33231771 PMC8354865

[19] Jepsen B Lomborg K Engberg M. GPs and involuntary admission: a qualitative study. Br J Gen Pract. (2010) 60:604–6. 10.3399/bjgp10X515115 20822693 PMC2913740

[20] Lyra RL de McKenzie SK Every-Palmer S Jenkin G. Occupational exposure to suicide: a review of research on the experiences of mental health professionals and first responders. PLoS ONE. (2021) 16:e0251038. 10.1371/journal.pone.0251038 33930087 PMC8087020

[21] Salza A Giusti L Ussorio D Casacchia M Roncone R. Cognitive behavioral therapy (CBT) anxiety management and reasoning bias modification in young adults with anxiety disorders: a real-world study of a therapist-assisted computerized (TACCBT) program vs. “person-to-person” group. CBT Internet Interv. (2020) 19:100305. 10.1016/j.invent.2020.100305 32055452 PMC7005461

[22] Fiol-DeRoque MA Serrano-Ripoll MJ Jiménez R Zamanillo-Campos R Yáñez-Juan AM Bennasar-Veny M. A mobile phone-based intervention to reduce mental health problems in health care workers during the COVID-19 pandemic (PsyCovidApp): randomized controlled trial. JMIR Mhealth Uhealth. (2021) 9:e27039. 10.2196/27039 33909587 PMC8133164

